# The Symbol Grounding Problem Revisited: A Thorough Evaluation of the ANS Mapping Account and the Proposal of an Alternative Account Based on Symbol–Symbol Associations

**DOI:** 10.3389/fpsyg.2016.01581

**Published:** 2016-10-13

**Authors:** Bert Reynvoet, Delphine Sasanguie

**Affiliations:** ^1^Brain and Cognition Research Unit, Faculty of Psychology and Educational SciencesKU Leuven, Leuven, Belgium; ^2^Faculty of Psychology and Educational SciencesKU Leuven Kulak, Kortrijk, Belgium

**Keywords:** numerical cognition, symbol grounding, symbol associations, approximate number system, mathematics achievement

## Abstract

Recently, a lot of studies in the domain of numerical cognition have been published demonstrating a robust association between numerical symbol processing and individual differences in mathematics achievement. Because numerical symbols are so important for mathematics achievement, many researchers want to provide an answer on the ‘symbol grounding problem,’ i.e., how does a symbol acquires its numerical meaning? The most popular account, the approximate number system (*ANS*) *mapping account*, assumes that a symbol acquires its numerical meaning by being mapped on a non-verbal and ANS. Here, we critically evaluate four arguments that are supposed to support this account, i.e., (1) there is an evolutionary system for approximate number processing, (2) non-symbolic and symbolic number processing show the same behavioral effects, (3) non-symbolic and symbolic numbers activate the same brain regions which are also involved in more advanced calculation and (4) non-symbolic comparison is related to the performance on symbolic mathematics achievement tasks. Based on this evaluation, we conclude that all of these arguments and consequently also the mapping account are questionable. Next we explored less popular alternative, where small numerical symbols are initially mapped on a precise representation and then, in combination with increasing knowledge of the counting list result in an independent and exact symbolic system based on order relations between symbols. We evaluate this account by reviewing evidence on order judgment tasks following the same four arguments. Although further research is necessary, the available evidence so far suggests that this *symbol–symbol association account* should be considered as a worthy alternative of how symbols acquire their meaning.

Previously, Feigenson et al. (2004) suggested that numerical symbols may be grounded in two core systems that are present in children from birth. First, numerical symbols (e.g., digits or number words) may be mapped onto a non-verbal and analog representation of numbers, typically referred to as the approximate number system (ANS). Second, numerical symbols may also be linked to a system that represents numbers in a precise way, but with a limited capacity up to 3–4. This system is sometimes referred to as the object tracking system (OTS) or parallel individuation system (e.g., Piazza, 2010). Although Feigenson et al. (2004) left open both possibilities, the past 10 years, a gradual shift toward the ANS as a solution for the symbol-grounding question has emerged. We will refer to this proposal as the *ANS mapping account* from now onwards (e.g., Piazza, 2010; Feigenson et al., 2013). Dehaene (2001) provided four arguments in favor of this ANS mapping account: First, approximate number processing abilities can be found in different species, ranging from bees, over fish to monkeys. Second, these approximate abilities are also present in infants. Third, similar behavioral patterns can be found in animals and infants on the one hand, and children and adults on the other hand, even when children and adults process symbolic numbers. Finally, there exist one dedicated brain region that is involved in non-symbolic and symbolic processing and calculation (an important subdomain of more complex mathematics achievement). When Halberda et al. (2008) for the first time observed that the performance in a non-symbolic number (arrays of dots) discrimination task - an ability relying on the ANS – was related to (symbolic) mathematics achievement, a fifth argument was added by the proponents of the ANS mapping account: The ANS serves as the foundational basis for more complex (symbolic) mathematical abilities, like for instance calculation.

In this paper, we will first critically evaluate these arguments on the basis of recent findings. Next, we will consider an alternative solution for the symbol-grounding problem, where the limited but precise number system (cf. the OTS, parallel individuation system or core system 2 of Feigenson et al., 2004) serves as a hub toward a representation of symbolic numbers where a numerical symbol derives its meaning through associations (e.g., order associations) with other symbols, an account we will refer to as the symbol–symbol association account. We will hereby review the same arguments that were raised in favor of the ANS mapping account, now applied to the alternative solution based on symbol–symbol associations: Evolutionary precursor of order, behavioral effects in ordering performance, dedicated brain area for ordering and calculation and the relation between ordering ability and mathematics achievement). Finally, we will sum up some conclusions and suggestions for further research to help unravel the symbol-grounding problem and consequently mathematics achievement.

## A Critical Evaluation of the ANS Mapping Account

As mentioned in the previous section, the ANS mapping account has traditionally been supported by five arguments. In our critical evaluation of these arguments, we group the first two of Dehaene (2001), i.e., approximate number processing can be found in animals and approximate number processing is present in infants. In this way, we critically evaluate the following four arguments in terms of their significance for the symbol grounding problem: (1) There is an evolutionary system underlying approximate number processing (evidenced by findings in animals and infants); (2) the same behavioral pattern is found in non-symbolic and symbolic processing; (3) there is a dedicated brain area that is activated by non-symbolic number, symbolic number and calculation and (4) non-symbolic processing is (predictively) related to mathematics achievement.

### Approximate Number Discrimination Is Present in Animals and in Infants

*First*, several studies have indeed shown that both animals (e.g., Cantlon and Brannon, 2006; Agrillo et al., 2011; Pahl et al., 2013) and infants (e.g., Xu and Spelke, 2000; Libertus and Brannon, 2010; Starr et al., 2013) can discriminate number. Crucially, the performance on these number discrimination tasks was suggested to rely on an imprecise representation of number. For instance, Cantlon and Brannon (2006) demonstrated that monkeys showed ratio dependent behavior: numerically closer numbers were more difficult to discriminate. Similarly, in a study with 6-month old infants, Libertus and Brannon (2010) demonstrated that the preference for the numerically changing stream (compared with the non-changing stream) increased when the ratio between the numbers increased. Such performance is supported by the idea of an ANS that represents number in an approximate and compressed way on a mental number line, resulting in the observation that numbers can only be correctly discriminated if they differ by a given numerical ratio (e.g., Dehaene, 2001). This idea has received further support by a series of single cell recording studies in monkeys by Nieder and Dehaene (2009) and Nieder (2016). They demonstrated the existence of ‘number neurons’ that encode number. More specifically, these number neurons showed maximum activity for a particular number (i.e., the preferred numerosity) and a gradual decrease of activity was observed in these neurons that was dependent on the ratio between the presented numerosity and the preferred numerosity.

Although the presence of basic numerical abilities in animals and infants is fascinating, most of these results are not informative regarding the question how symbols acquire their numerical meaning, i.e., the so-called ‘symbol grounding problem.’ However, the acquisition of the numerical meaning of symbols was the object of investigation in two studies by Diester and Nieder (2007, 2010). In both studies, monkeys were trained to associate numerical symbols with varying non-symbolic numbers. After the learning process, association neurons were found in the prefrontal cortex that responded similarly to the associated symbolic and non-symbolic number. Such association neurons, however, were not found in the parietal cortex, the subsumed dedicated brain area for number processing as we will describe later (Diester and Nieder, 2007). In another study, Diester and Nieder (2010) found that the monkeys’ behavioral performance with learned numerical symbols was similar to their performance with non-symbolic numbers. At first sight, these findings suggest that symbols acquire their numerical meaning by associating them with approximate magnitudes (i.e., the ANS mapping account). However, there is of course a crucial difference between training studies with monkeys and how children actually learn symbols during development. Before children grasp the numerical meaning of the symbols (i.e., the verbal number words), they have already acquired the numerical sequence of these verbal number words through recitation of the counting list (Wynn, 1990; Le Corre et al., 2006; Le Corre and Carey, 2007). This means that the meaning of numerical symbols can not only be inferred from their corresponding number of objects they are mapped on, but also from their relation with other numerical symbols (e.g., seven is larger than six because it comes later in the sequence). Because this language facility is absent in monkeys, it remains questionable whether the findings from training studies with monkeys can be generalized to how children acquire the numerical meaning of symbols.

### Non-symbolic and Symbolic Number Discrimination Show the Same Behavioral Effects

A *second* argument that is typically made to support the ANS mapping account is that children’s and adults’ performance in non-symbolic (i.e., deciding which of two presented dot arrays contains more dots), but crucially also in symbolic number comparison (i.e., deciding which of two digits is numerically larger) highly mirrors the behavioral patterns observed in infants and animals. Indeed, as in infants and animals, non-symbolic number comparison in children (e.g., Halberda et al., 2008; Holloway and Ansari, 2008; Sasanguie et al., 2013a) and adults (e.g., Buckley and Gillman, 1974; Halberda et al., 2012; Smets et al., 2016) is also characterized by a ratio effect (RE): participants are less accurate and respond slower when the numerical ratio between those numbers approaches 1 (comparing12 and 16 dots is for instance more difficult than comparing 8 and 16 dots). Also in symbolic comparison tasks similar effects can be found in children (e.g., Holloway and Ansari, 2008; Sasanguie et al., 2012) and adults (e.g., Moyer and Landauer, 1967; Dehaene et al., 1990): First, a distance effect (DE) is observed: deciding whether 7 is larger than 6 (i.e., small distance of 1) takes longer and is more difficult than deciding whether 7 is larger than 4 (large distance of 3). Second, a size effect (SE) is typically found (e.g., Dehaene and Mehler, 1992; Verguts et al., 2005): deciding whether 7 is larger than 6 (i.e., large numbers) is more difficult than deciding whether 3 is larger than 2 (i.e., small numbers). The combination of the distance and size effect mirrors the ratio effect in non-symbolic comparison tasks and is typically interpreted as evidence in favor of the ANS mapping account, i.e., the idea that symbols such as number words and digits acquire their numerical meaning by being mapped on the ANS, resulting in similar effects in non-symbolic and symbolic comparison.

However, already during a decade, Verguts et al. (2005), Van Opstal and Verguts (2011) and Verguts and Van Opstal (2014) showed by computational modeling work that overlapping approximate representations are not required to obtain a DE and a size effect and that these effects can be the result of simple network properties. Verguts et al. (2005) demonstrated that the DE in comparison can be caused by the connection weights between the input layer and the decision layer. This finding has been confirmed by the presence of a DE in non-numerical order processing where there is no approximate representational overlap between adjacent elements, such as navy ranks or academic positions (Chiao et al., 2004) or letters of the alphabet (Van Opstal and Verguts, 2011). In a more recent paper, Verguts and Van Opstal (2014) showed that the size effect in symbolic comparison is the result of the skewed frequency distribution of numbers, with smaller numbers being more frequent than larger numbers (Dehaene and Mehler, 1992): model simulations with equal frequencies for all elements did not lead to a size effect, whereas in contrast simulations with skewed frequencies did. The fact that the distance and size effect in symbolic comparison are not necessarily evidence in favor of overlapping representations and can be the result of decisional aspects and frequency distributions, respectively, clearly undermines the argument that similar behavioral effects such as the DE and SE in non-symbolic comparison tasks are evidence for ANS mapping account. Therefore, ideally, alternative tasks should be used to assess similarities in the behavioral performance with non-symbolic and symbolic numbers. One such task is the numerical matching task in which participants have to indicate whether two magnitudes are numerically similar or not (Cohen Kadosh et al., 2008; Van Opstal and Verguts, 2011). For instance, Sasanguie et al. (2015) presented participants with audio–visual number pairs that had to be numerically matched (i.e., are the number that you hear and the number that you see numerically equivalent or not?). When participants had to match a symbolic pair (i.e., a visual presented digit with an auditory presented number word), no DE was observed. In contrast, when a non-symbolic pair (i.e., an array of dots and auditory presented series of tones) or a mixed pair (i.e., an array of dots and an auditory presented number word) had to be matched, a DE was observed. Crucially, the performance on non-symbolic and mixed conditions was correlated, whereas no such correlation was observed with the symbolic condition. This finding is difficult to reconcile with the existence of one shared representation for number (i.e., the ANS on which numerical symbols are mapped). Rather, it suggests the existence of two different systems for processing non-symbolic and symbolic magnitudes. A similar conclusion was proposed by Lyons et al. (2012) on the basis of a different experimental manipulation. In this study, participants again had to discriminate pure symbolic, pure non-symbolic, and mixed pairs. It was hypothesized that if non-symbolic and symbolic numbers share the same representation, then mixed trials should be compared as efficiently as pure trials. Contrary to this hypothesis, comparing mixed trials occurred much slower. According to the authors, this additional cost reflects the time needed to connect two distinct magnitude representations for symbolic and non-symbolic number in order to solve the task. In sum, the modeling work and behavioral studies showed that we need to be cautious with the assumption that similar behavioral patterns for non-symbolic and symbolic number processing is evidence for the ANS account because (1) behavioral patterns observed for symbolic number processing can be explained alternatively and (2) when other measures than comparison are used, results contradicting the ANS mapping account have been obtained.

### Non-symbolic and Symbolic Number Discrimination Rely on the Same Brain Area

*A third* argument in favor of the ANS mapping account comes from neuroimaging studies. Several fMRI studies have shown that the parietal cortex and in particular the Intraparietal Sulcus (IPS), is activated during both non-symbolic and symbolic comparison suggesting that the IPS houses an abstract representation for both non-symbolic and symbolic number (for a review, see Nieder and Dehaene, 2009). Because the joint activation in IPS for non-symbolic and symbolic number processing can alternatively be explained by response processing activation, which also relies on parietal cortex (Göbel et al., 2004), more recent studies started to use more sensitive fMRI techniques with passive viewing conditions, like fMRI adaptation (e.g., Piazza et al., 2007; Notebaert et al., 2011; Vogel et al., 2014). For example, Piazza et al. (2007) habituated participants to either small or large, non-symbolic or symbolic numbers. After a period of habituation to the same stimulus, deviants could be presented that differed in number and/or notation. The authors observed a cross-notation adaptation in the IPS, reflected by a sustained decrease of the fMRI signal when a deviant was presented that was numerically close to the adapted number, irrespective of notation change. In contrast, no adaptation was found when the deviant was far away from the adapted number. This finding suggests that the IPS houses a representation of magnitude that is shared by non-symbolic and symbolic numbers. Another recent approach in fMRI research is the use of multivariate analytic techniques, a set of methods typically referred to as multi-voxel pattern analysis (MVPA). In contrast to more classic techniques in which activation is averaged across voxels, these new techniques analyze the pattern of activation of multiple voxels as a result of the presentation of a stimulus (e.g., a dot array of four) and relate it with the pattern of activation resulting from another stimulus (e.g., the symbol ‘4’) (for a review, see Eger, 2016). Using this approach, Eger et al. (2009) demonstrated that patterns of activation in the parietal cortex from digits could be used to predict the activation of the corresponding non-symbolic numbers. These results favor the idea that non-symbolic and symbolic number are represented by the same neurons located in the parietal cortex. Interestingly, this brain area is also one of the most consistently activated brain areas during arithmetic (for a meta-analysis, see Arsalidou and Taylor, 2011), confirming the idea that this abstract representation is crucial for mathematics achievement.

Although this might seem like a consistent story, several recent studies showed, however, results that do not easily fit into this account. For instance, Cohen Kadosh et al. (2011) reported the absence of cross-notation adaptation from non-symbolic to symbolic number, in contrast to Piazza et al. (2007). Contrary to what would be predicted on the basis of the ANS mapping account, Cohen Kadosh et al. (2011) found that parietal brain areas did not respond to changes in number and were only sensitive to changes in stimulus notation. Also studies using MVPA resulted in inconsistent findings and accordingly, provided counterevidence for a shared representation. In contrast to Eger et al. (2009) and Bulthé et al. (2014) were not able to find evidence for a shared distributed representation of number. Similarly, on the basis of a representational similarity analysis, Lyons et al. (2015) also observed no relation between the activity for a symbolic number and the activity evoked by the corresponding non-symbolic number, suggesting different representations for non-symbolic and symbolic numbers. However, these sophisticated fMRI techniques failing to find evidence for a shared representation for non-symbolic and symbolic numbers, cannot provide a strong test against the ANS mapping account. In their computational model, Verguts and Fias (2004) simulated numerical symbol learning by simultaneously presenting a symbolic and non-symbolic number. They demonstrated that the same neuronal layer that already represented non-symbolic numbers, also represented the newly learned symbolic numbers. However, the numerical symbols only partially recycled the representations of non-symbolic numbers and are more precisely represented than the non-symbolic numbers. As a result, this computational model showed that, although symbolic numbers are mapped on non-symbolic numbers, they don’t have identical representations. Therefore, the ANS mapping account should be evaluated further with other neuroimaging techniques as well. For example, using transcranial magnetic stimulation (TMS) – a neuroscience technique that can address causality – Sasanguie et al. (2013b) observed effects that are not consistent with the ANS mapping account. More specifically, they showed that the magnitude processing of symbolic numbers is not impaired after left and right parietal stimulation. In contrast, when a numerical symbol had to be associated with a non-symbolic stimulus, left parietal stimulation interferes with processing. Based on these results, the authors argued that symbolic numbers possibly can be processed outside the parietal cortex but that the parietal cortex remains important as an interface between non-symbolic and symbolic stimuli. In other words, symbolic numbers can be mapped or associated with non-symbolic numbers when appropriate for the task, but this mapping is not *per se* a prerequisite for all symbolic number processing tasks. In line with the idea that symbolic number processing can occur outside the IPS, Price and Ansari (2011) observed that the left angular gyrus was more activated during the presentation of Arabic numerals compared to both letters and scrambled stimuli. Because the left angular gyrus is also active during retrieval of arithmetic facts (e.g., Grabner et al., 2007), Price and Ansari (2011) suggested that this brain region is doing more than visual processing alone and is possibly involved in the initial mapping of symbols to their numerical meaning (Ansari, 2015). In sum, based on the studies reviewed above, there is clearly no consensus on (1) whether non-symbolic and symbolic numbers activate the same representation and (2) what exactly is the role of the IPS in case of symbolic number processing.

### Approximate Number Discrimination Is Related to Math Achievement

*Fourth*, and starting with the study of Halberda et al. (2008) demonstrating that the performance of 14-year old children in a non-symbolic comparison task was related to these children’s past scores on a (symbolic) mathematics achievement test, the argument was made that the observation that ‘individual differences in mathematics achievement are related to differences in the ANS’ supports the idea that ‘numerical symbols – which form in turn the basis of more complex mathematics achievement – are mapped onto an evolutionary ancient non-symbolic number representation.’ In the years to follow, this relation between non-symbolic number comparison and math achievement has been replicated several times in cross-sectional and longitudinal studies in children (e.g., Mundy and Gilmore, 2009; Libertus et al., 2011; Starr et al., 2013) and adults (e.g., Lyons and Beilock, 2011; Lourenco et al., 2012). In line with this, Piazza et al. (2010) observed that 10-year old children with a math learning disorder (i.e., developmental dyscalculia) have a less precise ANS compared to typically developing controls (see also Mazzocco et al., 2011 for similar findings in 14–15 year olds with dyscalculia). Recently, training studies in children (Hyde et al., 2014; Kuhn and Holling, 2014) and in adults (Park and Brannon, 2013, 2014) have confirmed the causal relation between the ANS and mathematics achievement by demonstrating that training on a non-symbolic comparison task resulted in improvement on a symbolic math achievement test.

However, again, the relationship between non-symbolic comparison and mathematics achievement was not consistently found (for reviews, see De Smedt et al., 2013 and Gebuis and Reynvoet, 2015). Several studies with typically developing children (e.g., Holloway and Ansari, 2009; Soltesz et al., 2010; Sasanguie et al., 2013a), adults (e.g., Inglis et al., 2011) and children with dyscalculia (e.g., Rousselle and Noël, 2007; De Smedt and Gilmore, 2011) reported no relation between non-symbolic number comparison and mathematics achievement. To address these inconsistencies, several groups conducted a meta-analysis (Chen and Li, 2014; Fazio et al., 2014; Schneider et al., 2016). These studies showed that the average association between non-symbolic processing and mathematics achievement is statistically robust, but relatively small (*r* between 0.20 and 0.24) and as a consequence, only can explain a small proportion of the explained variance in mathematics achievement. Moreover, recently, several studies have proposed that the observed association between non-symbolic number processing and mathematics achievement might be due to inhibitory processes (e.g., Fuhs and McNeil, 2013; Gilmore et al., 2013; Szűcs et al., 2013; Bugden and Ansari, 2016; for an overview see Leibovich and Ansari, 2016). Without any control for continuous magnitude features (total occupied area, individual dot size, density, …), number and these continuous features of a dot array are always highly correlated, just like number processing in daily life. For instance, the number of students in a class and the total area occupied by them are correlated: the more students, the more space that is occupied. To ensure that participants perform on the basis of number and do not use these continuous magnitude features, researchers have tried to control these continuous dimensions, by creating in half of the trials congruent stimulus pairs (i.e., continuous dimensions co-vary with number) and in the other half, incongruent stimulus pairs (i.e., continuous dimensions do not co-vary with number) (e.g., Piazza et al., 2007; Halberda et al., 2008; Gebuis and Reynvoet, 2011). Despite this control, several studies showed that continuous features still affect performance resulting in a congruency effect in a non-symbolic number processing task (e.g., Gebuis and Reynvoet, 2012; Leibovich and Henik, 2013, 2014; Szűcs et al., 2013; Leibovich et al., 2016b; Smets et al., 2016). Continuous magnitude features provide an additional cue for the correct response on congruent trials, but need to be inhibited on incongruent trials to arrive to a correct response. This inhibitory process results in slower and more erroneous responses on incongruent trials. More importantly, recent studies have now shown that this inhibitory process is responsible for the association between non-symbolic number comparison and mathematics achievement. Fuhs and McNeil (2013) for instance observed as the first that the relationship between the performance on a non-symbolic comparison task and arithmetic was mediated by inhibitory control. In line with this, Gilmore et al. (2013) demonstrated that the relation between the performance on non-symbolic comparison and arithmetic is purely driven by the performance on the incongruent trials on which inhibition is required to provide the correct response. Also more recently, Bugden and Ansari (2016) observed that the difference in performance between dyscalculic children and typically developing controls on a non-symbolic comparison task was mainly driven by performance differences in the incongruent trials, again indicating that not so much number processing but inhibitory processes are responsible for previously observed differences between both groups. However, inhibitory processes did not always accounted for the observed association between non-symbolic comparison and mathematics achievement (Keller and Libertus, 2015; Bellon et al., 2016). For instance, Keller and Libertus (2015) found that after controlling for the performance on a inhibitory control task, the relation between non-symbolic comparison and mathematics achievement persisted. Possibly, these inconsistent findings are due to different types of inhibitory tasks that were used because inhibition is not a unitary concept (Diamond, 2013) and to different protocols for creating the non-symbolic stimuli. It may be that some methods for creating non-symbolic stimuli may need more inhibition than others (see Clayton et al., 2015; Smets et al., 2016; DeWind and Brannon, 2016 for crucial differences between stimulus sets). These outstanding issues should be investigated further. To sum up, with regards to the fourth argument, we can conclude that the association between non-symbolic number processing and mathematics achievement is small (accounting for about 4–5% of the variance in mathematics achievement) and may be (partially) explained by inhibition. When also other domain-general processes – that tend to be understudied (e.g., Fias et al., 2013 for a similar claim) – should be controlled for, the association between non-symbolic processing and mathematics achievement may be vanished completely.

## An Alternative for the ANS Mapping Account: Symbol–Symbol Associations

After critically reviewing the main arguments in favor of the ANS mapping account, it is clear that this account cannot fully explain the symbol grounding problem, and consequently also does not provide us with a satisfying answer to the question what is so important/foundational for (future) math achievement? Therefore, in the next section, we argue that an alternative account based on symbol–symbol associations might bring solace in this regard. Crucially, our arguments to do so are very similar to the ones that have been put forward in favor of the ANS mapping account.

Before starting to review these arguments, it is crucial to pinpoint what exactly we refer to with the alternative account based on symbol–symbol associations. For this, it is necessary to go back in time, as we do not claim to propose something new here, but want to draw researchers’ attention to our remix of some old ideas. In contrast to the idea that children learn the numerical meaning of symbolic numbers by mapping them onto the ANS, the alternative account suggests that symbolic and non-symbolic numbers activate distinct representations (see e.g., Noël and Rousselle, 2011; Sasanguie et al., 2014). Concrete, symbolic numbers do not acquire their numerical meaning through a mapping process onto the ANS, but through the development of a new, more precise representation (Sasanguie et al., 2015). Whereas proponents of this account do not reject the existence of the ANS *per se* (although it can indeed be questioned whether this system should be kept referred to as the ANS or rather as some type of approximate magnitude system – AMS – see Leibovich et al., 2016a), they simply do not focus on it, because according to the alternative account, not the ANS but the other core system (cf. core system 2 as described by Feigenson et al., 2004 or the OTS or parallel individuation system as described by Piazza et al., 2010) is the hub for the emergence of an exact, separate symbolic system. Indeed, already 15 years ago, Carey (2001) proposed that the understanding of our initial exact numerical symbols (i.e., the verbal number words) relies on their association/mapping with the OTS, a system that allows us to keep track of up to four objects. Instead of being mapped onto the ANS, this account thus argues that initial numerical symbols (number words) are mapped onto the OTS (see also Benoit et al., 2004; Sarnecka and Lee, 2009; Slusser et al., 2013). According to Carey’s (2001, 2004, 2009) developmental model, next, the combination of these associations between the small symbolic numbers and the OTS and the increasing knowledge of the counting list (e.g., learned by means of songs, …. at home or in preschool) are used to infer critical principles of the numerical system, such as ‘order’ (i.e., numbers form sequences) and ‘the successor function’ (i.e., the next number is exactly one more than the previous one). Gradually, these principles are then applied to larger symbolic numbers, resulting in a complete understanding of the symbolic numerical system. As a result, (larger) symbolic numbers are primarily represented through order associations with other symbolic numbers (see also Nieder, 2009) instead of by their relations with their non-symbolic counterparts as proposed by the ANS mapping account. Recently, direct evidence for these symbol–symbol associations (without a link to the ANS) was provided by Davidson et al. (2012) who showed that 3–5 years old children could compare verbal number words larger than four, even they only knew the order between the verbal number words, but not their cardinal value, i.e., as measured with an estimation task in which the children had to produce the number word corresponding to a set of dots they had to estimate. Although there is quite some work on how young children map verbal number words to non-symbolic number, there is currently not much known about how other numerical symbols, like Arabic digits acquire their meaning. One possibility could be that digits are directly associated to the already known corresponding verbal numbers, resulting in similar associations between digits as the ones between the verbal numbers. Another possibility is that digits are, just like verbal numbers, first mapped onto the OTS and follow a similar trajectory as that of verbal numbers. To our knowledge, there is currently only one study that addressed the ability to map non-symbolic numbers, verbal numbers and digits in young children (3–5 years old). In this study, Benoit et al. (2013) observed that verbal numbers first are mapped on the OTS, followed by the mapping of digits onto the OTS. Only in a later phase a mapping between verbal numbers and digits occurs. Certainly, further research is necessary to examine the early development of digits, but the findings of Benoit et al. (2013) may suggest a delayed but similar developmental pattern for digits than for verbal numbers. Initially, they are mapped on the OTS and later, associations between the digits emerge when these digits are being connected to their verbal numbers that are part of the counting sequence. In sum, ‘the alternative account for the symbol grounding problem’ thus refers to the fact that over development, the ‘numerical meaning’ which a symbol must acquire, is reinterpreted: Whereas referring to the underlying non-symbolic and precise representation of small numbers (OTS) very early in development, with age, this numerical meaning gradually changes toward referring to its ordinal relation with other numerical symbols. Evidently, this alternative account based on symbol–symbol associations for the symbol-grounding problem has serious implications for studies investigating math learning and math performance because it seems very plausible that not the relation between symbols and their magnitudes (i.e., the ANS), but the relation between culturally acquired and language-facilitated symbolic numbers is foundational for future math achievement and thus responsible for individual differences in this regard.

To date, the domain of numerical cognition has been largely dominated by the ANS mapping account, resulting in a lack of studies seriously investigating the alternative account of an independent symbolic number system based on symbol–symbol associations. Moreover, also with regards to research focusing on the basis of individual differences in mathematics achievement, the insufficient exploration of this alternative may have led to an incomplete understanding of the contribution of domain-specific skills underlying math learning and performance. Recently, however, more and more studies started to use order judgment tasks instead of comparison (for a review, see Lyons et al., 2016). In a typical order judgment task, participants have to decide whether a pair or a triplet of (symbolic or non-symbolic) numbers is presented in the correct order (e.g., ascending from left-to-right) or not. Clearly, such a task addresses symbol–symbol associations instead of symbol-magnitude relations (which is the case in comparison, cf. the ANS mapping account). Reviewing these recent studies, we argue that the symbol–symbol associations (instead of the symbol-magnitude relations, cf. the ANS mapping account) are crucial for (future) mathematics achievement. We will support this idea with four arguments which are very similar to those that have been put forward in favor of the ANS mapping account.

### Order Processing Is Present in Animals and Infants

*First*, studies with animals (e.g., Brannon and Terrace, 1998) and infants (e.g., Suanda et al., 2008; Picozzi et al., 2010; for a review, see Sury and Rubinsten, 2012) have shown that, when dot arrays are presented, not only the quantity of the arrays (i.e., how many) but also the order between the quantities (i.e., increasing or decreasing) can be processed. For instance, Brannon and Terrace (1998) demonstrated that monkeys can learn the order of quantities, even when extended to new quantities. Suanda et al. (2008) showed that 11-month old infants were sensitive to order relations between three quantities. More specifically, when the infants were habituated to three quantities in an increasing order, they looked longer at test trials when the order was reversed (i.e., descending order). In contrast 9-month old infants were only sensitive to order relations when other variables like area and size co-varied with number, which seem to suggest that the number dimension cannot be processed in isolation from other continuous variables in 9-month old children. In sum, there seems to be an evolutionary precursor for order processing. However, as we already indicated before, the ability to understand order relations between non-symbolic quantities (next to cardinal aspects) in animals and infants is not a guarantee that to be acquired symbolic orders built upon these innate abilities. For learning symbolic orders, like the counting list or the alphabet, language plays an important role. Children learn for instance order relations between numbers and between letters through games and songs and as a consequence, form order relations between these stimuli based on associations without being aware of the exact meaning of the stimuli (Wynn, 1990; Le Corre et al., 2006). Therefore the absence of language abilities in animals and infants, limits these studies in their generalizability to how older children acquire the meaning of symbolic numbers.

### Non-symbolic and Symbolic Order Processing Show Different Behavioral Effects

*Second*, as explained above, this alternative proposal for the symbol grounding problem assumes the development of an exact separate symbolic system. As a consequence, different behavioral patterns can be expected in non-symbolic and symbolic number processing tasks (see for instance Sasanguie et al., 2015). Also in numerical ordering tasks, different effects have been observed for non-symbolic and symbolic number. When participants have to decide whether three non-symbolic dot arrays are in increasing or decreasing order, a classic DE appears: Slower reaction times are observed when the numerical distance between the dot patterns is small, suggesting the same mechanisms play a role as in a comparison task (Lyons and Beilock, 2013; Rubinsten et al., 2013). By contrast, when the same decision has to be made on triplets of digits, a reversed DE is found: Reaction times are faster when the numerical distance between the digits is small (e.g., Franklin et al., 2009; Rubinsten and Sury, 2011; Lyons and Beilock, 2013; Lyons et al., 2014). In sum, although the theories about which exact (cognitive) mechanisms underlie this reversed DE remain relatively speculative so far (Franklin et al., 2009), the different behavioral effects (i.e., canonical/standard DE vs. reversed DE)clearly show that order is processed differently in non-symbolic and symbolic numbers.

### Non-symbolic and Symbolic Order Processing Rely on Different Brain Areas

A *third* argument for this alternative account about how symbols are grounded is that symbolic order processing activates different brain areas than non-symbolic order processing, suggesting that the processing of symbolic number relies on (partially) different circuits. In a neuroimaging study by Lyons and Beilock (2013) contrasting directly non-symbolic and symbolic order processing of numbers, it was demonstrated that the parietal cortex was important for non-symbolic order processing, but not for symbolic order processing. In contrast, symbolic order processing resulted in activation of the left premotor cortex. This finding suggests that different mechanisms might be responsible for non-symbolic and symbolic order processing. The authors suggested the parietal activation in the non-symbolic condition was a consequence of multiple comparisons based on quantity. To decide whether three dot patterns are in increasing order, one has to compare the quantity of first two dot arrays, followed by a comparison of the quantity of the last two dot arrays. The premotor activation in the symbolic order condition is according to the authors due to the result of retrieval of a highly rehearsed list of stimuli, like the count-list. Interestingly, activation in premotor cortex has also been observed during simple additions (Pesenti et al., 2000; Knops and Willmes, 2014) indicating that the brain area that possibly houses order associations between numbers, is also active during more complex calculation. However, the majority of studies focused exclusively on symbolic ordering and hereby observed IPS activation suggesting that also order and not just quantity is processed by the IPS (Fias et al., 2007; Franklin and Jonides, 2008; Kaufmann et al., 2009; Zorzi et al., 2010; Attout et al., 2014). At present, it is therefore still unclear what exactly the functional role of the parietal and premotor cortex is and how they interact in symbolic order processing (Ansari, 2015; Lyons et al., 2016). One possible explanation to account for these inconsistencies is the divergence in tasks to address order processing. Some studies ask participants to decide which of two items comes before or after in a sequence and as a consequence, can possibly be resolved through comparison: to decide whether 7 comes after 4, participants can rely on quantity (e.g., Fias et al., 2007). Other studies presented triplets of digits (e.g., Lyons and Beilock, 2013). Because of the presence of a robust reversed DE in the ordering task with triplets, this task more reliably suggests the activation of ordinal processing (Franklin et al., 2009; Lyons and Beilock, 2011, 2013). In sum, although the only study that contrasted non-symbolic and symbolic order processing so far (Lyons and Beilock, 2013) does suggest involvement of different brain areas, the limited number of imaging studies addressing order processing makes clear that the functions of the different brain areas underlying order processing need to fine-tuned.

### Symbolic Order Processing Is Related to Math Achievement

*A fourth argument* we want to make is that the performance in an order judgment task is related to mathematics achievement: Efficient processing of order is associated with higher mathematics achievement. This relation has been observed in typically developing children (e.g., Lyons et al., 2014; Lyons and Ansari, 2015), in studies with dyscalculic children (Rubinsten and Sury, 2011; Attout and Majerus, 2015) and in adults (Lyons and Beilock, 2011; Goffin and Ansari, 2016). For instance, in a large scale study by Lyons et al. (2014), it was shown that order processing was the best predictor of mathematics achievement from third grade onwards. In younger children, symbolic comparison was the best predictor. Although this need to be confirmed with additional studies, such a finding is perfectly in line with the alternative account we propose here for numerical symbol grounding where symbols are first mapped onto (a precise) representation of quantity and then, through development are being more and more associated with one another. Crucially, and inconsistent with a classic ANS mapping account, the unique contribution of the non-symbolic comparison task was never statistically reliable. In line with this, Attout and Majerus (2015) compared the performance of a group of children with developmental dyscalculia with typically developing controls matched for age, language abilities and IQ. These authors found that dyscalculic children were slower on non-symbolic and symbolic ordering tasks. In contrast, no slowing was observed on non-symbolic and symbolic comparison tasks, indicating that order (and not quantity) may be responsible for the mathematical problems observed in dyscalculia. To summarize, the growing amount of studies investigating order processing have now clearly shown that symbolic order performance is related to mathematics achievement. More importantly, the observed associations are much larger than the associations that are typically observed between non-symbolic number processing and mathematics achievement. This may be the result of the fact that (symbolic) mathematics achievement is building more upon symbol–symbol associations, that are addressed in judgements about order, than symbol-magnitude associations as has been traditionally assumed.

## Conclusion

In this review we addressed the highly important symbol grounding question for numerical symbols. Feigenson et al. (2004) suggested that our understanding of symbols might be rooted into two independent core systems: an imprecise ANS and a precise system for a small number of objects. The past decade, the idea that symbols derive their meaning by being associated to the ANS, i.e., the ANS mapping account, became far more popular than its alternative. In this review, we critically evaluated four arguments for the dominant ANS mapping account that were raised on the basis of previous findings (Dehaene, 2001; Halberda et al., 2008). This evaluation made clear that the arguments are less strong than sometimes believed. Therefore, we argued for an alternative account (based on a remix of old ideas of e.g., Carey, 2001 and Nieder, 2009, see further), where the initial (small) symbols (i.e., number words) might be first mapped onto a precise representation (i.e., the OTS) and in combination with inferring principles about relations (e.g., the successor function) and language (e.g., counting routines) result in a separate system for symbolic number where numerical symbols are represented through associations which each other. In fact, the developmental trajectory based on Carey (2001) we proposed here, is also not very different from other proposals, like the one from Nieder (2009). He describes the human development of symbol processing as going from an indexical (i.e., associations between symbols and the corresponding number of objects) to a symbolic stage (i.e., associations between symbols or the syntax of symbols). The main point we want to make here is that the latter stage did not receive as much attention as the first stage, but on the other hand, is probably the most important one for (future) mathematics achievement. We evaluated this alternative account on the basis of four arguments, parallel to the ones used in favor of the ANS mapping account. By means of this review, we want to convince the reader that symbol–symbol associations should be considered as a worthy alternative for the ANS mapping account, which has been dominating the numerical cognition research to date. Of course more evidence from future studies is needed, but the available evidence for this very plausible alternative account and for its implications with regard to math achievement so far already seem promising.

## Author Contributions

All authors listed, have made equal and substantial, direct and intellectual contribution to the work, and approved it for publication.

## Conflict of Interest Statement

The authors declare that the research was conducted in the absence of any commercial or financial relationships that could be construed as a potential conflict of interest.
